# Supplementary material for: Targeting key angiogenic pathways with a bispecific CrossMAb optimized for neovascular eye diseases
Source: EMBO Mol Med. 2016 Oct 14;8(11):1265–88. doi: 10.15252/emmm.201505889 (PMC5090659; doi:10.15252/emmm.201505889)

Appendix table of Content

1) **Appendix Material &Methods**
2) **Appendix Figure Legends S1-4**
3) **Appendix Figures S1-4**

1) Appendix Material &Methods

Neonatal C57Bl/6J mice were exposed to hyperoxia at day 7 (P7) for a period of 5 days. Mice were subsequently returned to normoxic condition and antibodies were administered on day 14 (P14) by intraperitoneal (IP) injection at 10 mg/kg. Mice were sacrificed at day 17 (P17). Retinal whole mounts were prepared for immunohistochemistry against isolectin GS-IB4 Alexa Fluor 594 conjugate (Invitrogen, Life Technologies) to detect retinal vasculature. DAPI (4',6-diamidino-2-phenylindole, dihydrochloride; Life Technologies) was used to label cell nuclei as counterstain. Stained retinal whole mounts were imaged using a Zeiss LSM700 confocal microscope (Carl Zeiss AG, Germany) and avascular areas and neovascularization (neovascular tufts) were analyzed. The experiment was performed at an ophthalmic contract research organization: Experimentica Ltd, Kuopio, Finland according to the ARVO statement of use of animal in ophthalmic and vision research and the EC directive EC86/609EEC for animal experimentation and the protocol approved by the animal experimentation board of Finland.

Endotoxin induced uveitis (EIU) was induced in C57BL/6 mice by a single intraperitoneal injection of 7.5 mg/kg lipopolysaccharide (LPS). Antibodies were administered one day prior to EIU induction intraperitoneally at 10 mg/kg. Then, 24 h after LPS administration, retinal leukocyte infiltrations were evaluated by counting of cross sections. Aqueous humor was obtained by puncturing the cornea using a 29 gauge needle at 24 h after LPS injection. Cell content of aqueous humor was determined in each eye by pipetting two drops of 1 µl each on a poly-L-lysine-coated slide and cells counted.

Appendix Figure Legends S1-4

Appendix Figure Legend S1

Human endothelial cells were plated on filters and transendothelial resistance measured using CellZscope technology. After TEER values have reached its peak and stabilized 10 ng/ml of VEGF-A was and TEER values measured over time. After 18 h three concentrations of RG7716 were added to the culture wells and TEER value recording continued. SD of three experiments is shown in grey. Statistical analysis was performed for the final TEER values at the 42 h time point. ANOVA ($P < 0.0001$) and Dunnett's multiple comparisons were used with control group as comparator for n = 3 Experiments. Only VEGF-A treatment was significantly different to control, see asterisk, $P < 0.0001$. Addition of RG7716 fully reverted the VEGF-A induced TEER reductions.

Appendix Figure Legend S2

Comparison of IgG control, anti-VEGF and anti-VEGF-A/ANG-2 in the model oxygen induced retinopathy in the mouse (n = 10). A) Schematic presentation of the experimental design, mice were placed into hyperoxia at postnatal D7 for 7 days and normoxia from D14 for 3 days. At D14, mice received a single IP injection of 10 mg/kg of antibody. B,C) Assessment of the area of vascular obliteration and area of neovascularization after retinas were imaged using confocal microscope in retinal whole mount stained with isolectin GS-IB4 Alexa Fluor 594 conjugate. Error bars show SEM with ***$P < 0.001$, **$P < 0.01$ and *$P < 0.05$ using ANOVA (avascular: ***, $P = 0.0002$; neovascular tufts: ns ***, $P = 0.052$) and multiple t-test with n = 10 animals per group. In B) anti-VEGF-A/ANG-2 is significantly different from IgG control (***, $P = 0.0002$) and from anti-VEGF-A (**, $P = 0.0031$); in C) anti-VEGF-A/ANG-2 is significantly different from IgG control (*, $P = 0.0458$).

Appendix Figure Legend S3

Inhibition of leukocyte infiltration into the retina and aqueous by combined inhibition of VEGF-A and ANG-2 in the model of systemic LPS challenge. Antibody was given IP at 10 mg/kg 24 h before LPS challenge. A) Retinal cross section of IgG control and anti-VEGF-A/ANG-2-treated tissue stained with hematoxylin and eosin. Infiltrating leukocytes are observed in the inner plexiform layer, see arrows. B) Infiltrating cells were counted on retinal cross sections and numbers shown by bar diagram. C) Aqueous fluid was taken from animals and numbers of infiltrating leukocytes counted demonstrated by bar diagram. Error bars show SEM and all significant changes are shown with using ANOVA (both: $P < 0.0001$) and Tukey's multiple t-test with n = 8 animals per group. In B) challenge only and IgG control was significantly different vs untreated and CrossMAb anti-VEGF-A/ANG-2 (****, $P < 0.0001$) and anti-VEGF-A/ANG-2 combination (***, $P = 0.0003$, $P = 0.0001$, respectively). Furthermore anti-VEGF-A is different from CrossMAb anti-VEGF-A/ANG-2 (**, $P < 0.0086$) and anti-VEGF-A/ANG-2 combination (*, $P = 0.046$) and untreated (****, $P < 0.0001$). Finally anti-ANG-2 is different from untreated (***, $P = 0.001$). In C) untreated was different from IgG control (****, $P < 0.001$), anti-VEGF-A (***, $P = 0.0004$). and anti-ANG-2 (***, $P = 0.0004$). Furthermore CrossMAb anti-VEGF-A/ANG-2 was different from IgG control (***, $P = 0.0002$).

Appendix Figure Legend S4

Aqueous and serum concentrations of antibodies 1 day after intravitreal antibody delivery (D16) and at the end of the experiment (D30) in non-humane primate laser-induced CNV. A) Aqueous levels of all antibodies were determined by ELISA at D16 and D30. Comparison of treatment groups for each day did not reveal any significant concentration differences at D16 or D30 despite different antibody formats using using ANOVA and multiple *t*-test with n = 6 comparing the time points separately. B) Mean serum levels of all antibodies were determined at different time points after IVT delivery. IgG control and anti-ANG-2 (both wild-type IgG1 molecules) showed near-constant serum concentrations over time. CrossMAb RG7716 with modified Fc showed lower systemic concentrations. The Fab fragment ranibizumab showed the lowest serum concentrations for all time points measured.

Appendix Figure S1

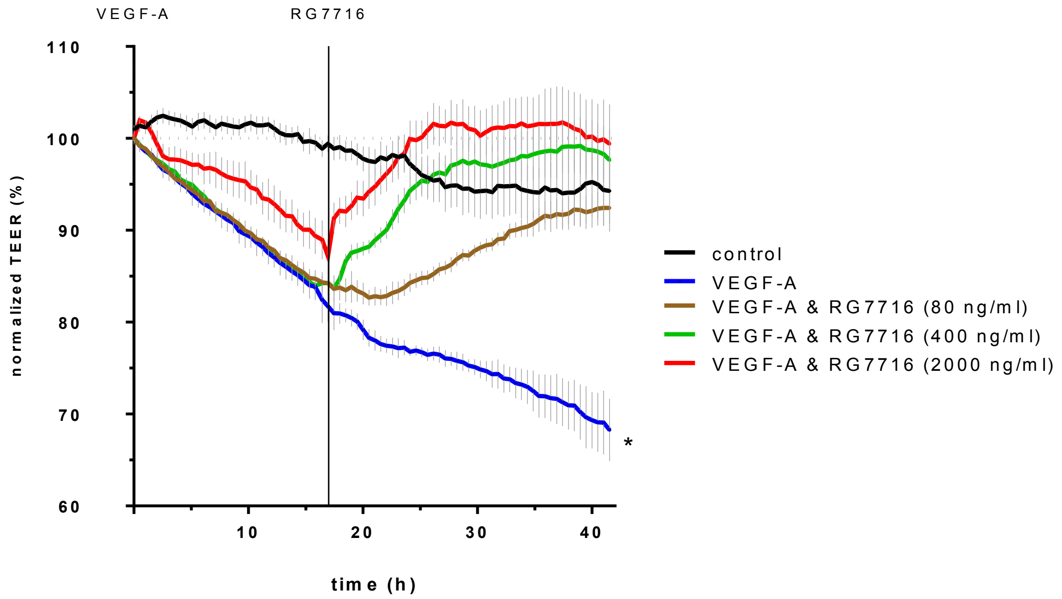

Appendix Figure S2

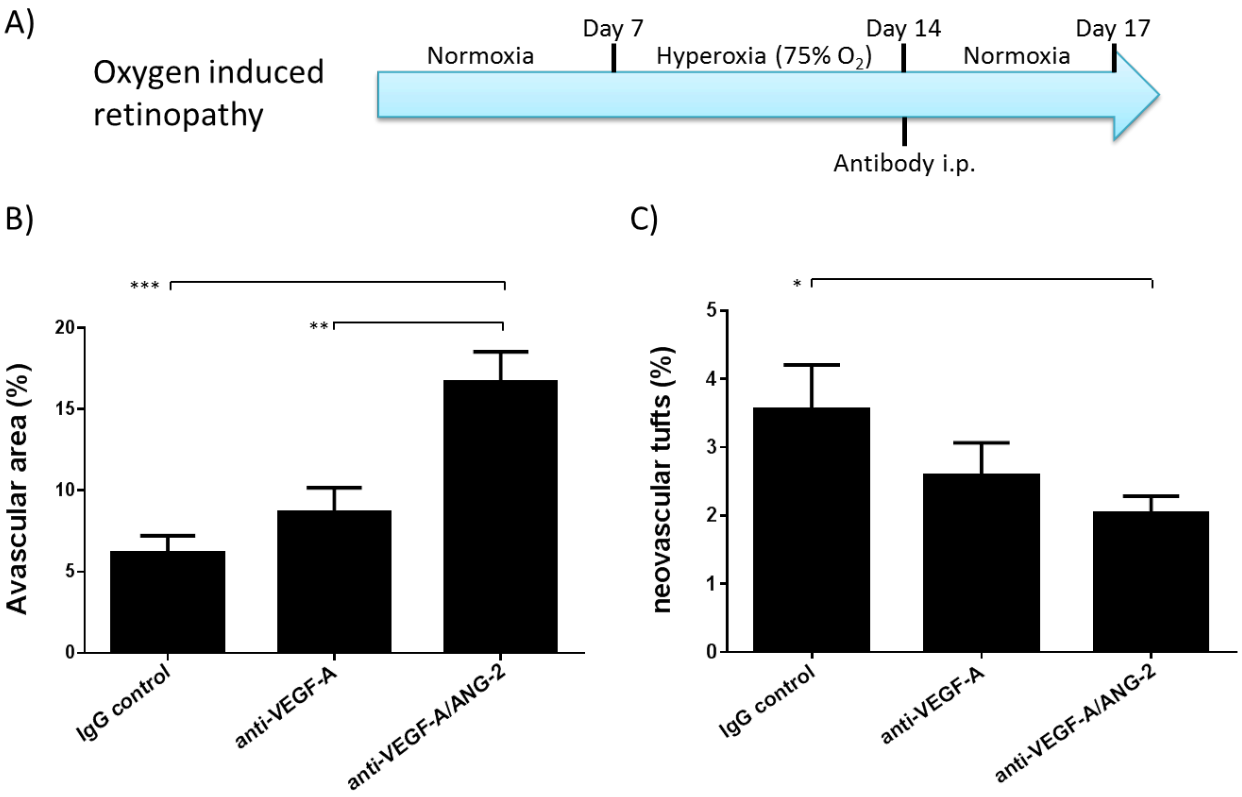

Appendix Figure S3

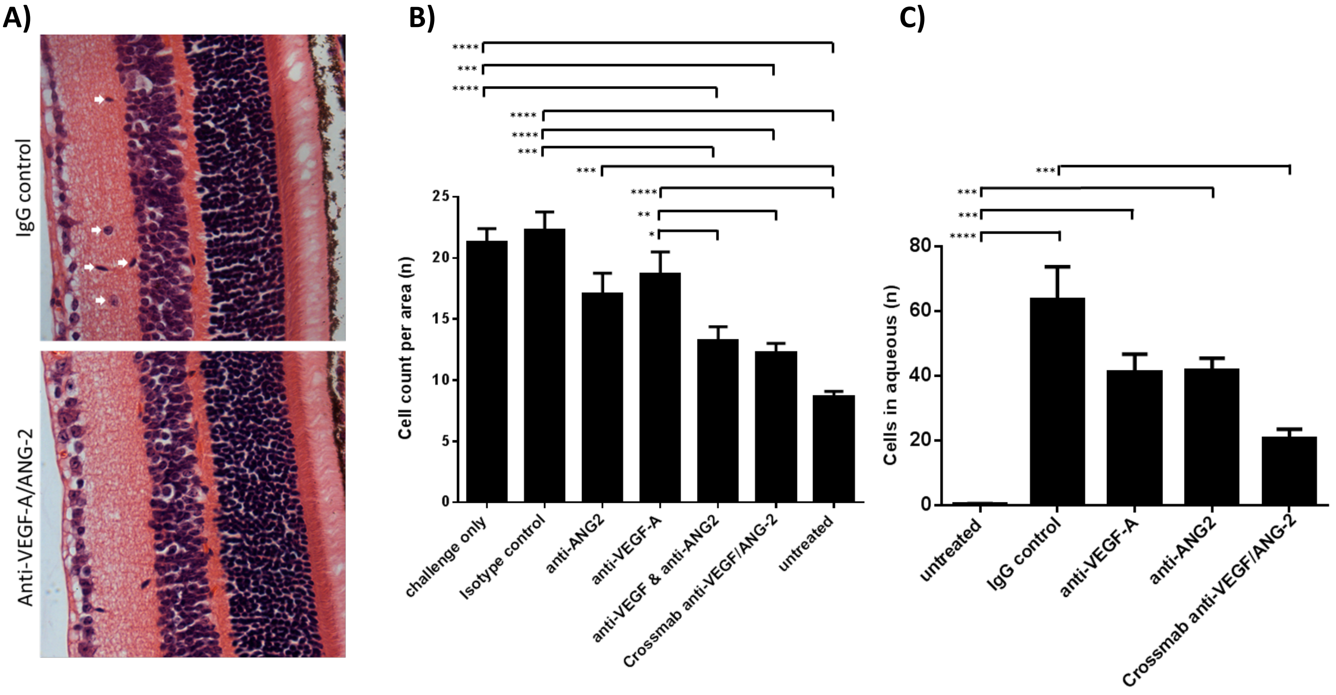

Appendix Figure S4

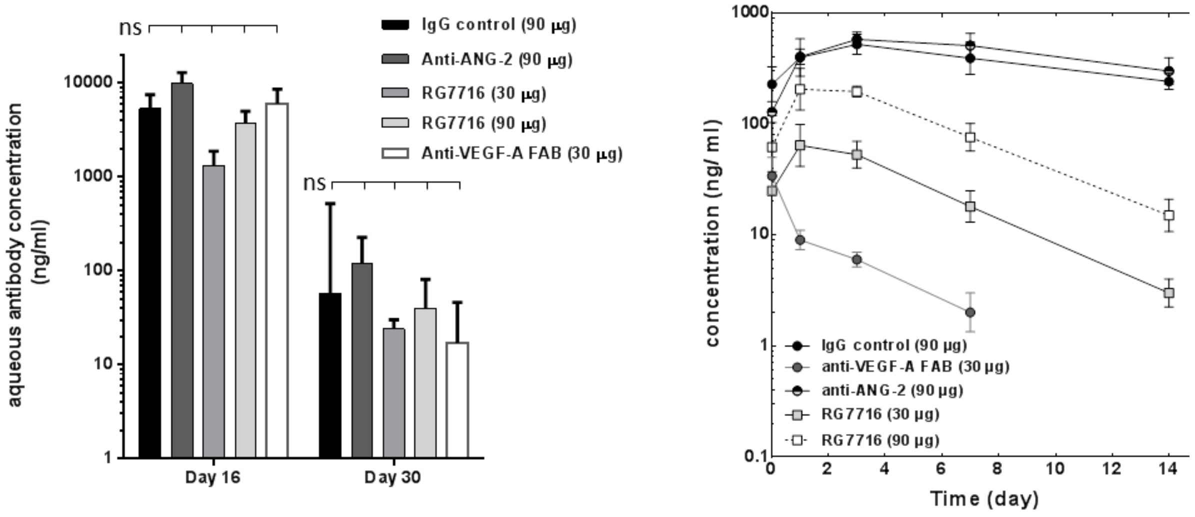

Supplement: Supplementary file 1 — Appendix [file EMMM-8-1265-s001.pdf]
